# Stochastic Dynamics of the COVID-19 Case-Fatality Ratios in Indonesia, Malaysia, and the Philippines: Economic Implications for the Post-COVID-19 Era

**DOI:** 10.3389/fpubh.2021.755047

**Published:** 2021-09-28

**Authors:** Zili Shi, Hua Zhang, Ren Zhang, Lili Zhu

**Affiliations:** ^1^School of International Business and Economics, Henan University of Economics and Law, Zhengzhou, China; ^2^School of Economics and Academy of Financial Research, Zhejiang University, Hangzhou, China; ^3^McCoy College of Business Administration, Texas State University, San Marcos, TX, United States; ^4^School of Business, Shenandoah University, Winchester, VA, United States

**Keywords:** COVID-19 pandemic, Case-Fatality Ratios, CFR, Indonesia, Malaysia, the Philippines

## Abstract

This paper analyses the stochastic dynamics of the COVID-19 Case-Fatality Ratios (CFR) in three developing economies in East Asia: Indonesia, Malaysia, and the Philippines. The sample covers the daily frequency data from April 28, 2020, to June 29, 2021. For this purpose, we utilize two unit root tests, which consider one structural break and two structural breaks. The findings reveal that the CFR follows a unit root process in Indonesia and the Philippines. However, the CFR is stationary in Malaysia. This evidence indicates that the COVID-19 has a permanent effect in Indonesia and the Philippines but temporary in Malaysia. The paper also discusses the potential economic implications of these results for the post-COVID-19 era in the related developing economies.

## Introduction

The COVID-19 pandemic started in China in December 2019 and has affected all regions of the World. However, due to the connectedness of the Eastern Asian economies to the Chinese economy, East Asian countries have been the first countries that have been affected by the COVID-19 pandemic (1). However, these countries have experience with regional pandemics, such as the Severe Acute Respiratory Syndrome (SARS) pandemic between 2002 and 2003 (2).

The spread out of the pandemics has been generally measured by the Case-Fatality ratio (henceforth CFR) in the empirical literature; how many people died due to the infectious disease relative to the total cases. This measure also represents the survival rate from an infectious disease (3). According to the Worldometers COVID-19 Coronavirus Pandemic data tracker, accessed on August 6, 2021, 202,319,670 people have been infected from the COVID-19 coronavirus 4,289,076 people have been died due to this new type of coronavirus (4). Therefore, the CFR of the COVID-19 pandemic can be calculated as 2.12% at the global level on a corresponding day. However, the CFR values of the COVID-19 across countries are quite heterogonous. Because of this issue, successful forecasts of the pandemic pattern are becoming a tough challenge (5).

Given these backdrops, this paper analyses the stochastic dynamics of the COVID-19 CFR values in three developing economies in East Asia: Indonesia, Malaysia, and the Philippines. These three developing economies had great potential for solid economic performance before the COVID-19 pandemic. Therefore, detecting the stochastic dynamics of the COVID-19 pandemic can be important for policymakers to reach their countries' economic potential before the pandemic. In addition, policymakers can think about stimulus packages for the negative consequences of the COVID-19 on economic indicators if there is a significant spillover effect.

Examining the stochastic process of the CFR of the COVID-19 can be crucial for policymakers and scholars. At this stage, if we observe that the CFR of the COVID-19 is stationary, policy implications (e.g., lockdowns) will have temporary effects on the CFR values. If the CFR of the COVID-19 follows a unit root, policy implications will have persistent effects to change the pattern of the CFR of the COVID-19. In addition, the stochastic process of the CFR of the COVID-19 can provide implications on spillover effects to macroeconomic variables. For instance, if the CFR value follows a unit root process, there can be significant spillovers to other economic variables, such as economic performance, inflation, employment levels, and small business revenues (6).

Furthermore, analyzing the stochastic process of the CFR of the COVID-19 is related to the predictability of the pattern of the pandemic. If the CFR value is stationary, it is possible to predict the future pattern of the COVID-19 pandemic in a given country. Finally, the stochastic process of the CFR can provide implications for econometric methodologies. For instance, if the CFR value follows a unit root process, the empirical analyses can be used with the cointegration and error correction models. However, if the CFR value is stationary, one should implement Autoregressive Distributed Lag (ARDL) or other traditional regression methods.

On the other hand, various papers have analyzed the determinants of the CFR of the COVID-19. Some of these papers, of course, have considered the stochastic dynamics of the CFR of the COVID-19. For instance, Diaz et al. (7) examine the dynamic characteristics of the COVID-19 pandemic in Colombia, measured by the CFR. The authors find that the pattern of the COVID-19 pandemic is predictable. The probability of death, the intensive-care unit admission, and the hospitalization rates were determined by age and sex. Li et al. (8) also show that the CFR of the COVID-19 pandemic is mainly related to age, according to the data from the regions of China. Sorci et al. (9) find that the CFR values significantly vary among the European countries. The authors suggest that the variation among the CFR values is temporary. Their findings show that age, democracy, per capita income, and unhealthy conditions increase the CFR values.

Moreover, Daw (10) shows that the conflicts raise the CFR values in Libya, Syria, and Yemen. Zhai et al. (11) further develop this evidence by using the cross-section data from 120 countries. Daw (10) and Zhai et al. (11) conclude that conflicts are the main variables determining the CFR. Khan et al. (12) also observe that the CFR of the COVID-19 is negatively related to the efficiency of government, civil society, and health expenditures using the dataset of 86 countries. Hradsky and Komarek (13) find that age, average temperature, gross domestic product, health conditions, health facilities, population density, and urban population are the main determinants of the variation of the CFR values across countries. These studies show that the CFR values of the COVID-19 are predictable across countries.

As we have discussed, suppose that the CFR value is found as stationary. In this case, policy implications for mitigating the spread out of the pandemic (e.g., lockdowns) will only have temporary effects. However, if the CFR values follow a unit root process, stringency measures (e.g., school closures and travel bans) will have persistent effects to change the pattern of the CFR values of COVID-19. Our paper tests the validity of the above hypothesis by utilizing two different unit root tests with one endogenous structural break (14) and two endogenous structural breaks (15) in the CFR values. Given that there are several waves in the COVID-19 pandemic, which is mostly related to the seasonal cycles, using more than one endogenous structural break in the CFR series can be noteworthy to capture the different dynamics of the pandemic. According to the findings of the empirical examinations, the CFR follows a unit root process in Indonesia and the Philippines. However, the CFR is stationary in Malaysia. This evidence indicates that the COVID-19 has a permanent effect in Indonesia and the Philippines but temporary in Malaysia.

The rest of the study is organized as follows. Section Data and Unit Root Test Methodology provides the details of the data and explains the unit root tests methodology. Section Empirical Findings discusses the empirical findings with their potential implications. Section Concluding Remarks presents the concluding remarks.

## Data and Unit Root Test Methodology

### Data

This paper focuses on the COVID-19 Case-Fatality Ratios (CFR) in three developing economies in East Asia: Indonesia, Malaysia and the Philippines. The sample covers the daily frequency data from April 28, 2020, to June 29, 2021. Following Khan et al. (12), the starting date of the data is based on a day, which had 1,000 confirmed cases at least in a country. The total number of observations is 428 for each country.

**Figure 1:**
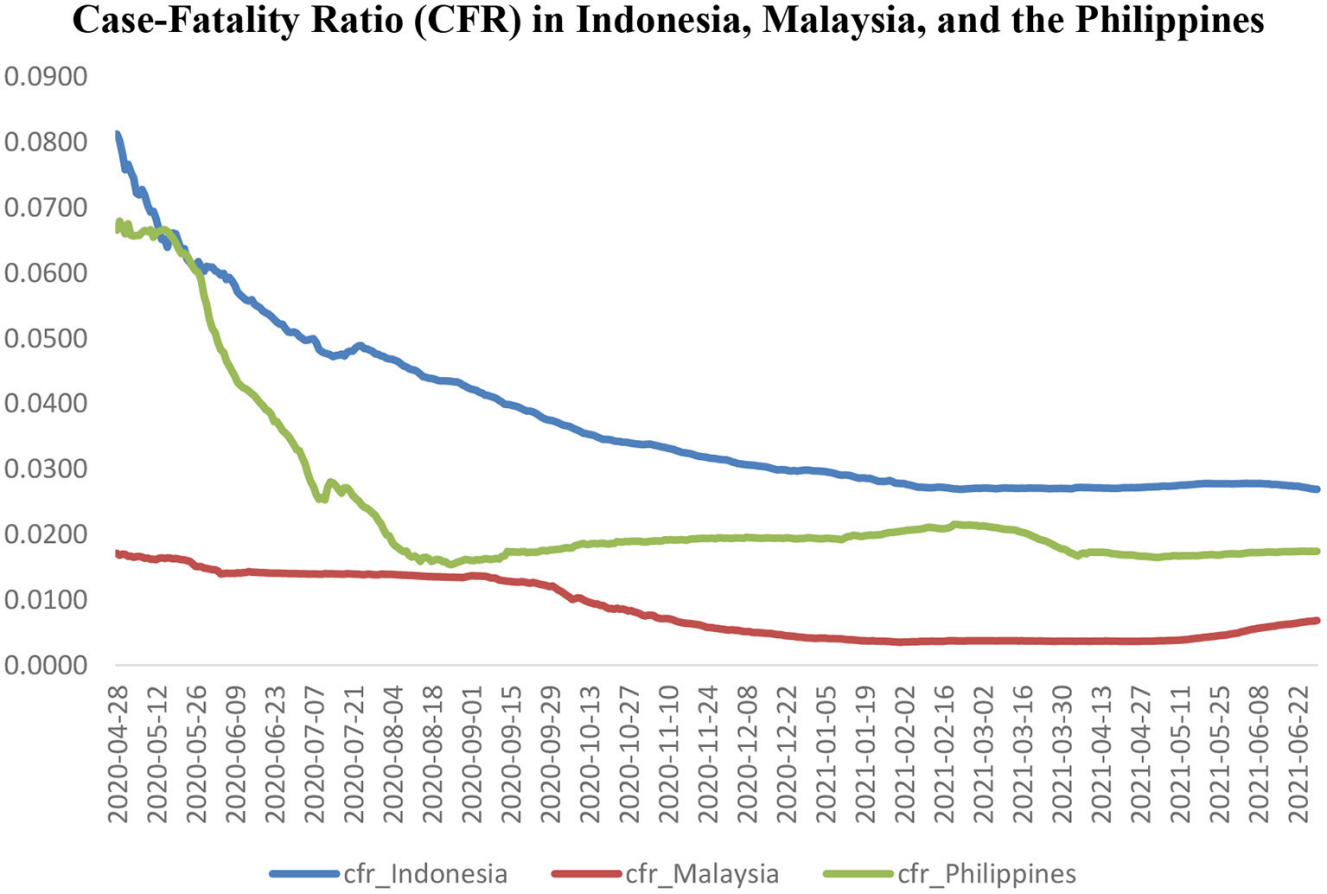
Case-Fatality Ratio (CFR) in Indonesia, Malaysia, and the Philippines.

Following previous papers [e.g., (16)], the CFR ratios are calculated as the total deaths related to the COVID-19, relative to the COVID-19 cases in a given country. We download the data for the COVID-19 cases and the deaths related to the COVID-19 from the *Cross-country Database of COVID-19 Testing*, introduced by Hasell et al. (17). In addition, the patterns of the COVID-19 Case-Fatality Ratios (CFR) in Indonesia, Malaysia and the Philippines are illustrated in Figure 1.

Figure 1 demonstrates a dramatic downward trend in the CFR ratio in Indonesia from April 2020 to March 2021. However, the decline of the CFR ratio was stabilized from March 2021 to June 2021 in the country. There was also a significant decline in the CFR ratio in Indonesia from April 2020 to January 2021. The period between January 2021 and May 2021 was a stable period regarding the CFR ratio in Malaysia. However, there was a significant rise in the CFR ratio in Malaysia from May 2021 to June 2021. In addition, the CFR ratio in the Philippines sharply decreased from April 2020 to September 2020. However, the CFR ratio in the Philippines gradually increased from September 2020 to March 2020. After a relative decline, the CFR ratio in the Philippines was stable from April 2020 to June 2020.

On the other hand, Table 1 summarizes the descriptive statistics of the CFR values of the COVID-19 in Indonesia, Malaysia, and the Philippines.

**Table 1:** Descriptive statistics.

**Country**	**Indicator**	**Source**	**Mean**	**Std. Dev**.	**Min**.	**Max**.	**Obs**.
Indonesia	CFR	Hasell et al. (17)	0.037	0.012	0.026	0.081	428
Malaysia	CFR	Hasell et al. (17)	0.008	0.004	0.003	0.017	428
Philippines	CFR	Hasell et al. (17)	0.024	0.013	0.015	0.067	428

The findings in Table 1 show that the CFR in Indonesia is the highest (3.7%), followed by the Philippines (2.4%). The lowest CFR is obtained in Malaysia (0.8%). The volatility of the CFR values, measured by standard deviations, are also significantly higher in Indonesia (1.2%) and the Philippines (1.3%) compared to Malaysia (0.4%). The peak of the CFR values during the period under concern is the highest in Indonesia (8.1%), followed by the Philippines (6.7%). The lowest peak value of the CFR is calculated in Malaysia (1.7%). The minimum values of the CFR are also similar to other measures: It is the highest in Indonesia (2.6%) and then followed by the Philippines (1.5%). The lowest minimum value of the CFR is observed in Malaysia (0.3%). These preliminary findings show that the CFR values of the COVID-19 pandemic are different in Malaysia compared to Indonesia and the Philippines.

### Econometric Methodology: Unit Root Tests With Endogenous Breaks

This paper implements two unit root tests with different structural breaks, proposed by Clemente et al. (15) and Zivot and Andrews (14). Firstly, we consider the unit root test of Zivot and Andrews (14), which accounts for one endogenous structural break in the CFR values. The unit root test methodology of Zivot and Andrews (14) focuses on one endogenous structural break and extends the unit root test of Perron (18). At this stage, the unit root test of Zivot and Andrews (14) suitably detects the break date; therefore, it is a more powerful unit root test than the unit root test of Perron (18). It is also important to note that the unit root test with an endogenous structural break can be more powerful than the unit root tests with no breaks for modeling the CFR of the COVID-19 values in different countries. At this stage, we focus on one break on the level of series. If we find the unit root, we also consider the one break on the first series difference. The null hypothesis of the unit root test of Zivot and Andrews (14) is that the CFR value in a given country follows a unit root process. The optimal number of lags is selected by the Bayesian Information Criteria (BIC). Besides, the maximum lags are 12, and the trimmer rate is 0.05. The critical values are provided by Zivot and Andrews (14).

However, the COVID pandemic patterns depend on different waves due to the nature of infectious diseases. Therefore, there can be more than one structural break in the CFR values of the COVID-19. Therefore, we utilize the unit root tests of Clemente et al. (15), which model two endogenous structural breaks in the CFR values. Using the unit root test with two structural breaks can increase the power of unit root tests. At this stage, we consider the level of the series with two breaks. If we find the unit root, we consider the one break on the first difference of the series. The null hypothesis of the unit root test of Clemente et al. (15) is that the CFR value in a given country follows a unit root process. We use the BIC method to select the optimal number of lags. The maximum lags are 12, and the trimmer rate is 0.05. The critical values are shown in Clemente et al. (15).

## Empirical Findings

Table 2 provides the findings of the unit root test of Zivot and Andrews (14).

**Table 2:** Unit root test of Zivot and Andrews (14).

**CFR**	**Test statistics**	**CV (1%)**	**CV (5%)**	**CV (10%)**	**Lag**	**Break date**	**Test statistics**	**CV (1%)**	**CV (5%)**	**CV (10%)**	**Lag**	**Break date**
Indonesia	−4.463	−5.34	−4.80	−4.58	4	28-08-2020	−8.925^***^	−5.34	−4.80	−4.58	3	11-07-2020
Malaysia	−5.907^***^	−5.34	−4.80	−4.58	4	01-10-2020	−7.688^***^	−5.34	−4.80	−4.58	4	04-09-2020
Philippines	−4.045	−5.34	−4.80	−4.58	4	21-10-2020	−7.007^***^	−5.34	−4.80	−4.58	2	09-07-2020

*The table reports (i) one break on the level of series (left column) and (ii) the one break on the first difference of series (right column)*.

*Null hypothesis: The indicator follows a unit root process. The optimal number of lags is selected by the BIC*.

*The maximum lags are 12. The Trimmer rate is 0.05. CV, Critical Value*.

*** *p < 0.01*.

Table 2 shows that the CFR values follow a unit root process in Indonesia and the Philippines. However, the CFR value in Malaysia is stationary. Note that one structural break in the level is used in the unit root test of Zivot and Andrews (14). When we consider the first difference of the series, all CFR values become stationary.

Table 3 reports the findings of the unit root test of Clemente et al. (15).

**Table 3:** Unit root test of Clemente et al. (15).

**CFR**	**Test statistics**	**CV (5%)**	**Lag**	**Break dates**	**Test statistics**	**CV (5%)**	**Lag**	**Break dates**
Indonesia	−3.529	−3.56	1	17-11-2020; 2021-06-08	−10.68^***^	−3.56	6	22-05-2020; 2021-12-01
Malaysia	−4.519^***^	−3.56	2	14-07-2020; 2020-10-01	−5.820^***^	−3.56	5	09-06-2020; 2021-01-16
Philippines	−3.095	−3.56	6	29-05-2020; 2021-13-03	−4.836^***^	−3.56	3	10-07-2020; 2020-22-09

*The table reports (i) two breaks on the level of series (left column) and (ii) the two breaks on the first difference of series (right column)*.

*Null hypothesis: The indicator follows a unit root process. The optimal number of lags is selected by the BIC*.

*The maximum lags are 12. The Trimmer rate is 0.05. CV, Critical Value*.

*** *p < 0.01*.

Table 3 indicates that CFR values follow a unit root process in Indonesia and the Philippines when two structural breaks in the level are considered. At this stage, the CFR value in Malaysia is stationary. Note that when we focus on the first difference of the series, all CFR values become stationary. The results from Malaysia, which show the predictability of the CFR values, are in line with the findings of Diaz et al. (7), Sorci et al. (9), Daw (10), Zhai et al. (11), Khan et al. (12), and Hradsky and Komarek (13). However, the findings from Indonesia and the Philippines, which provide the unpredictability of the CFR values, are in line with the previous evidence in Ioannidis et al. (5).

This evidence indicates that the COVID-19 has temporary effects on the CFR in Malaysia. Our findings provide several implications: Firstly, the CFR in Malaysia is found stationary. However, the CFR series follows a unit root process in Indonesia and the Philippines. Therefore, the COVID-19 has a permanent effect in Indonesia and the Philippines.

Secondly, in line with the previous empirical papers [e.g., (6, 19, 20)], there can be significant transmissions of external shocks from the CFR of the COVID-19 values to other economic variables (e.g., economic performance, employment, personal consumption, small business revenues, and small business openings) and they can be investigated in Indonesia and the Philippines. A proper time series technique can be the cointegration method and the error correction model in these countries. However, scholars can utilize the ARDL or other regression methods in Malaysia using the CFR values.

Thirdly, given that the CFR values follow a unit root process in Indonesia and the Philippines, it is not possible to predict the pattern of the COVID-19 pandemic using the CFR values in these countries. However, the pattern of the COVID-19 pandemic can be predicted in Malaysia using the CFR value in the country. This issue may be important when the CFR of the COVID-19 will be used as the dependent variable in the empirical analyses [see e.g., (10–12)].

## Concluding Remarks

This paper analyzes the stochastic dynamics of the daily CFR values of the COVID-19 pandemic in Indonesia, Malaysia and the Philippines from April 28, 2020, to June 29, 2021. To this end, we run the unit-root test with one structural break proposed by Zivot and Andrews (14) and the unit root with two structural breaks introduced by Clemente et al. (15). We observe that the CFR follows a unit root process in Indonesia and the Philippines. However, the CFR is stationary in Malaysia. The results show that the COVID-19 has a permanent effect in Indonesia and the Philippines; however, the impact is temporary in Malaysia. We also suggest that the unit root test with more than one break can capture several waves of the COVID-19 pandemic during the period under concern.

These results provide several economic implications for the post-COVID-19 era in these economies. For instance, external shocks during the COVID-19 pandemic have permanently affected the CFR values in Indonesia and the Philippines, which is significant evidence of the shifting business cycles. The high level and the persistent CFR levels in Indonesia and the Philippines cause limited mobility, which hurts macroeconomic activity in these countries. At this stage, direct income supports, reducing the household debt burden, monetary policy tools, and tax reliefs can be important policy implications to normalize the devastating effects of the COVID-19 crisis in Indonesia and the Philippines. The results also indicate that the CFR levels are significantly different in the countries in the same region with a similar per capita income level. This evidence supports the hypothesis that countries have been differently affected by the COVID-19 pandemic.

Future papers can analyze the stochastic dynamics of the COVID-19 related indicators in other countries by using different time-series and panel data methods. At this stage, the econometric techniques, which consider more than one structural break, can better capture the several waves and different variants of the COVID-19 pandemic.

## Data Availability Statement

Publicly available datasets were analyzed in this study. This data can be found here: https://ourworldindata.org/coronavirus-testing.

## Author Contributions

ZS: empirical estimations and paper writing. HZ: data collection and paper writing. RZ: methodology and paper writing. LZ: supervision and paper writing. All authors contributed to the article and approved the submitted version.

## Funding

The authors acknowledge the funding from the Philosophy & Social Science Fund of Tianjin City, China. Award #: TJYJ20-012 (Prompting the Market Power of Tianjin City's E-commerce Firms in Belt & Road Countries: A Home Market Effect Approach).

## Conflict of Interest

The authors declare that the research was conducted in the absence of any commercial or financial relationships that could be construed as a potential conflict of interest.

## Publisher's Note

All claims expressed in this article are solely those of the authors and do not necessarily represent those of their affiliated organizations, or those of the publisher, the editors and the reviewers. Any product that may be evaluated in this article, or claim that may be made by its manufacturer, is not guaranteed or endorsed by the publisher.
